# Hyperpigmentation with Capecitabine: Part of Hand-Foot Syndrome or a Separate Entity?

**DOI:** 10.7759/cureus.2397

**Published:** 2018-03-30

**Authors:** Jennifer Caprez, Umar Rahim, Amna Ansari, Muhammad Uzair Lodhi, Mustafa Rahim

**Affiliations:** 1 Department of Medicine, Appalachian Regional Healthcare, Beckley, Lincoln Memorial University-Debusk College of Osteopathic Medicine; 2 Pre-Medical Student, Department of Sciences, Queens University of Charlotte, Nc; 3 Medicine, Mcmaster University Michael G. Degroote School of Medicine, Canada; 4 Medical Student, Department of Medicine, Raleigh General Hospital, Beckley, Wv; 5 Assistant Clinical Professor of Internal Medicine, West Virginia University School of Medicine

**Keywords:** capecitabine induced hyperpigmentation, hand-foot syndrome

## Abstract

Capecitabine has several side effects, most common of which is Hand-Foot syndrome (HFS); however, less frequently reported is capecitabine-associated hyperpigmentation. The hyperpigmentation associated with this drug has been documented to involve the hands and feet and, less commonly, the mucous membranes of the mouth. To our knowledge, it has never been documented to involve the face. We report a case of a patient with capecitabine-induced Hand-Foot Syndrome (HFS), who also presented with hyperpigmentation of the hands, feet, oral mucosa, and face.

## Introduction

Capecitabine is an oral chemotherapy medication, used most frequently for breast and colorectal cancer, taken at home twice daily for the first two weeks of a three-week cycle. This medication is a prodrug of 5-fluorouracil (5-FU), and it is converted to 5-FU by thymidine phosphorylase, which is an enzyme found in high levels in tumor cells. This conversion to the active metabolite by thymidine phosphorylase increases the ability of the drug to target cancer. Even though capecitabine spares the majority of the surrounding healthy tissue, it is still known to cause HFS. HFS was first described in patients taking mitotane. Since then, it has been associated with other chemotherapy agents, including anthracyclines, taxanes, cytarabine, methotrexate, cyclophosphamide, fluoropyrimidines, and capecitabine [1].

## Case presentation

**Figure 1:**
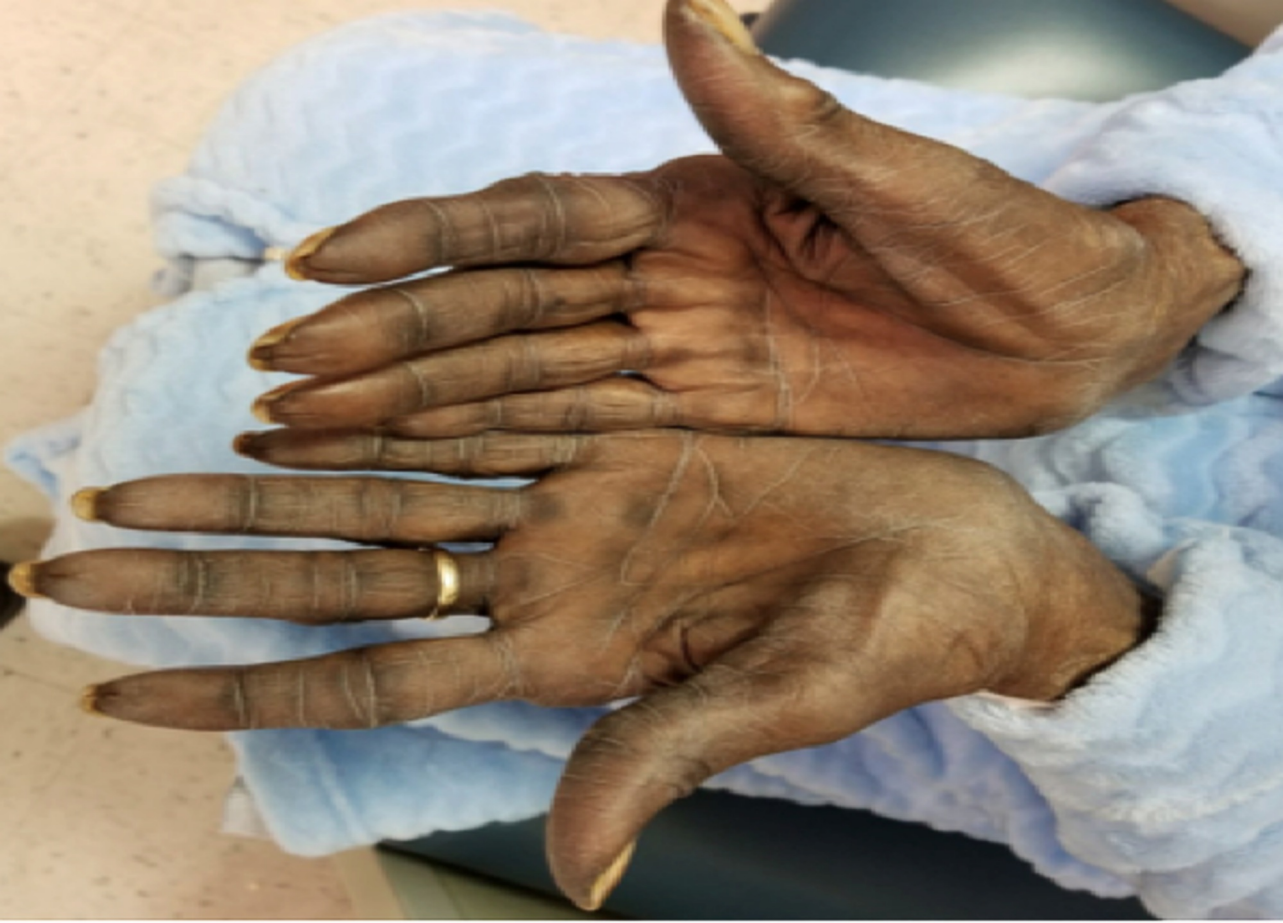
Physical exam of the hands showing dry skin and mild discoloration, two weeks after initiating capecitabine.

The patient was an 80-year-old African American female with a history of rectal cancer being treated with capecitabine. During her office visit recently, she was found to have an incidental finding of dry, peeling skin with mild discoloration of her hands (Figure 1). The patient denied any pain, swelling, numbness, or paresthesias.

**Figure 2:**
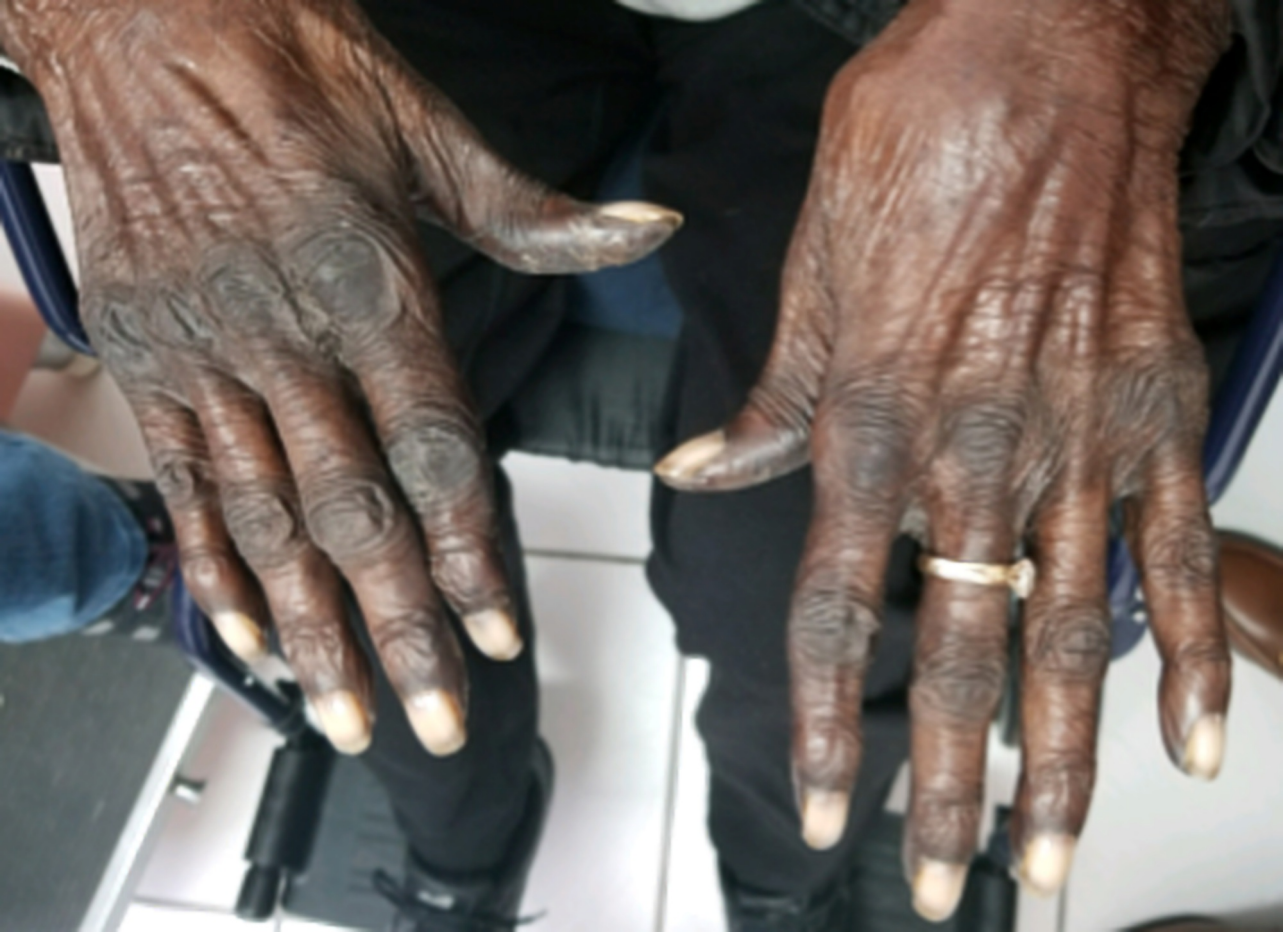
Physical exam of the hands showing markedly increased discoloration of the dorsal surface, eight weeks after the initiation of capecitabine.

**Figure 3:**
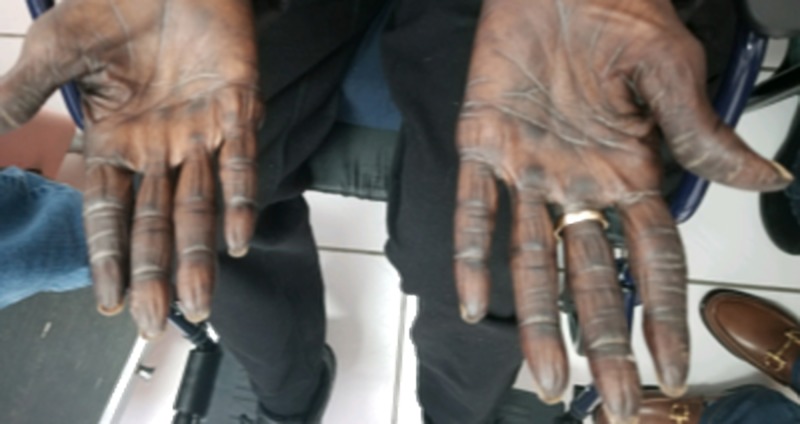
Physical exam of the hands showing markedly increased discoloration of the palmar surface, eight weeks after the initiation of capecitabine.

Six weeks later, at follow-up, this patient was seen with a markedly increased discoloration of her hands, which now also involved her feet, oral mucosa, and face. Especially, the hands showed a significant increase in discoloration, which included both the dorsal and palmar surfaces (Figures 2-3). 

**Figure 4:**
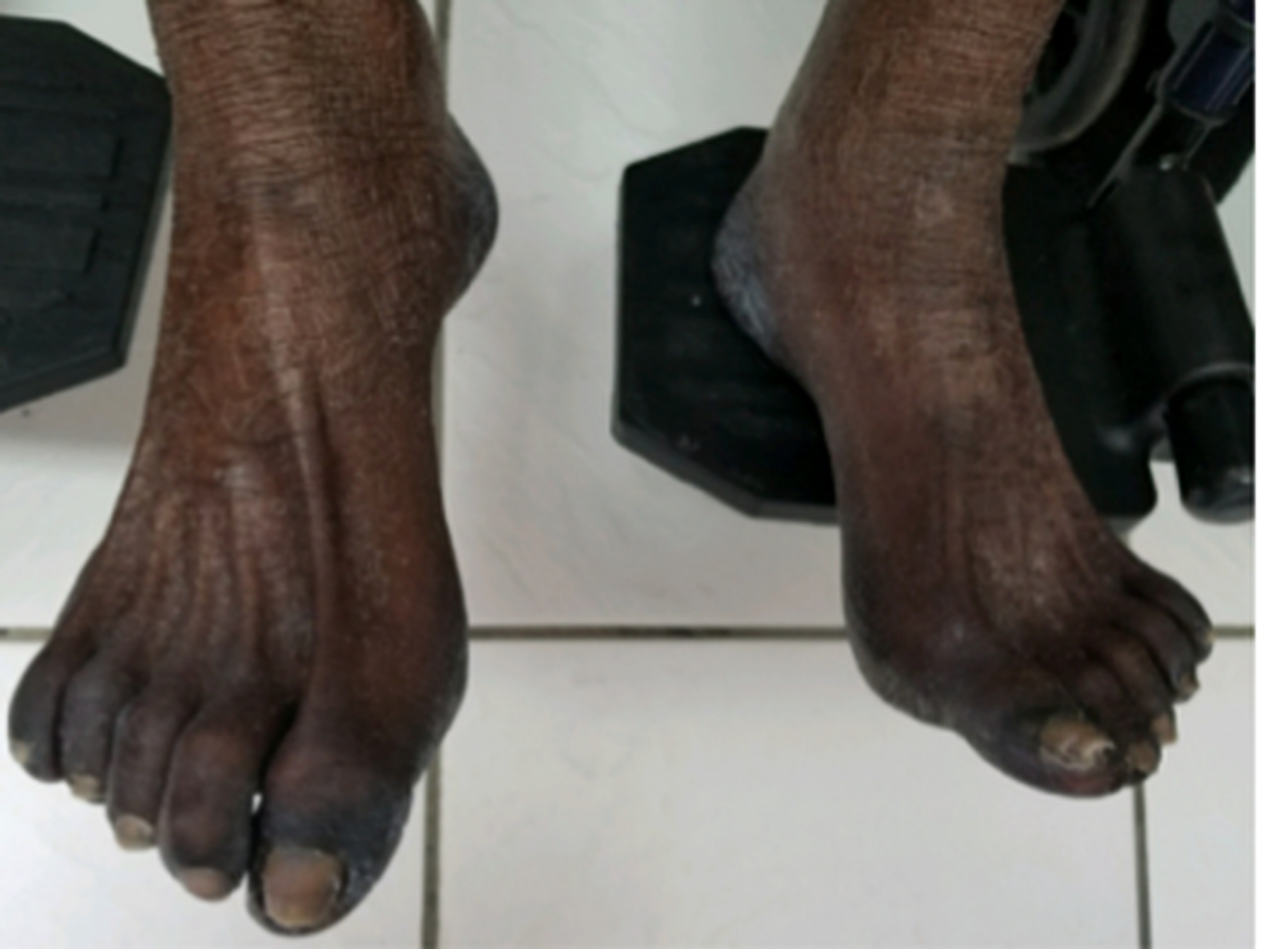
Physical exam of the feet showing marked discoloration of the soles and dorsal surface.

The patient’s feet also showed discoloration, which was worst around the toes (Figure 4).

**Figure 5:**
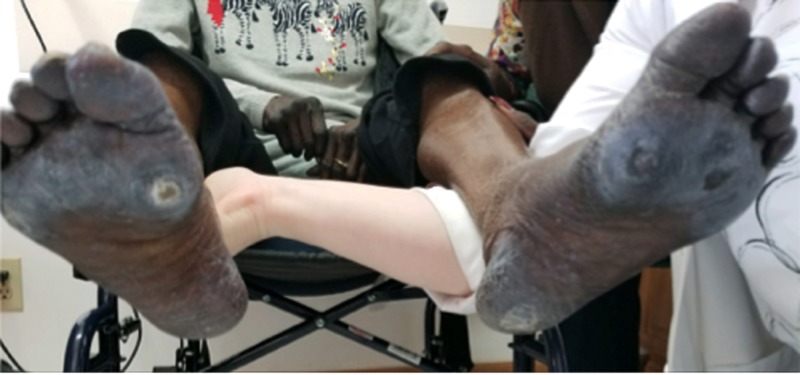
Physical exam of the feet showing dry and cracked skin with an ulceration just proximal to the first metatarsophalangeal joint, bilaterally. Note: The white discoloration of the feet is caused by the lotion used by the patient.

​​​​​​The soles of the feet also had ulcerations just proximal to the first metatarsophalangeal joint, bilaterally (Figure 5).

**Figure 6:**
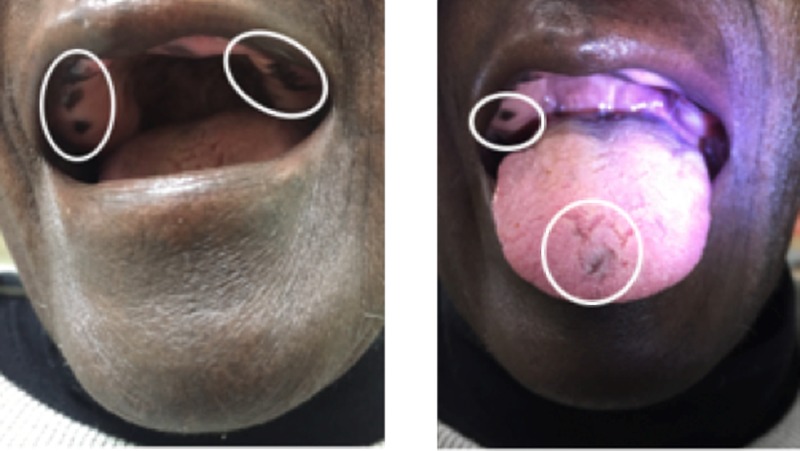
Physical exam of the oral cavity showing several areas of hyperpigmentation involving the upper and lower gums, as well as the tongue. Note: The patient is edentulous.

Areas of discoloration in the patient’s oral mucosa, specifically the upper and lower gums and the tongue, were found as well. The patient was edentulous, and the gum discoloration seemed to be in the areas where her teeth would have been (Figure 6).

**Figure 7:**
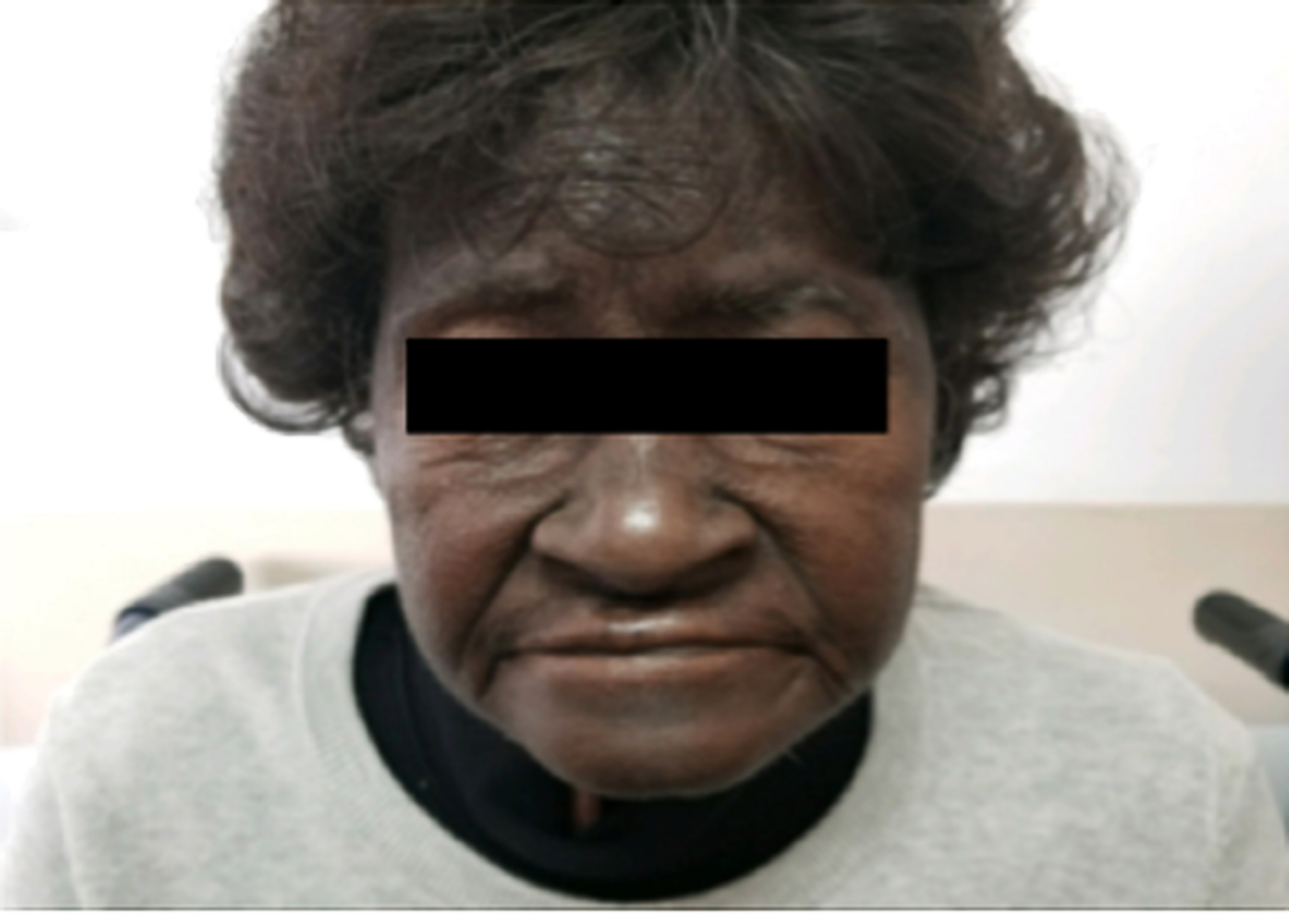
Picture showing marked discoloration of the forehead and the skin surrounding the nose, several weeks after the treatment with capecitabine.

**Figure 8:**
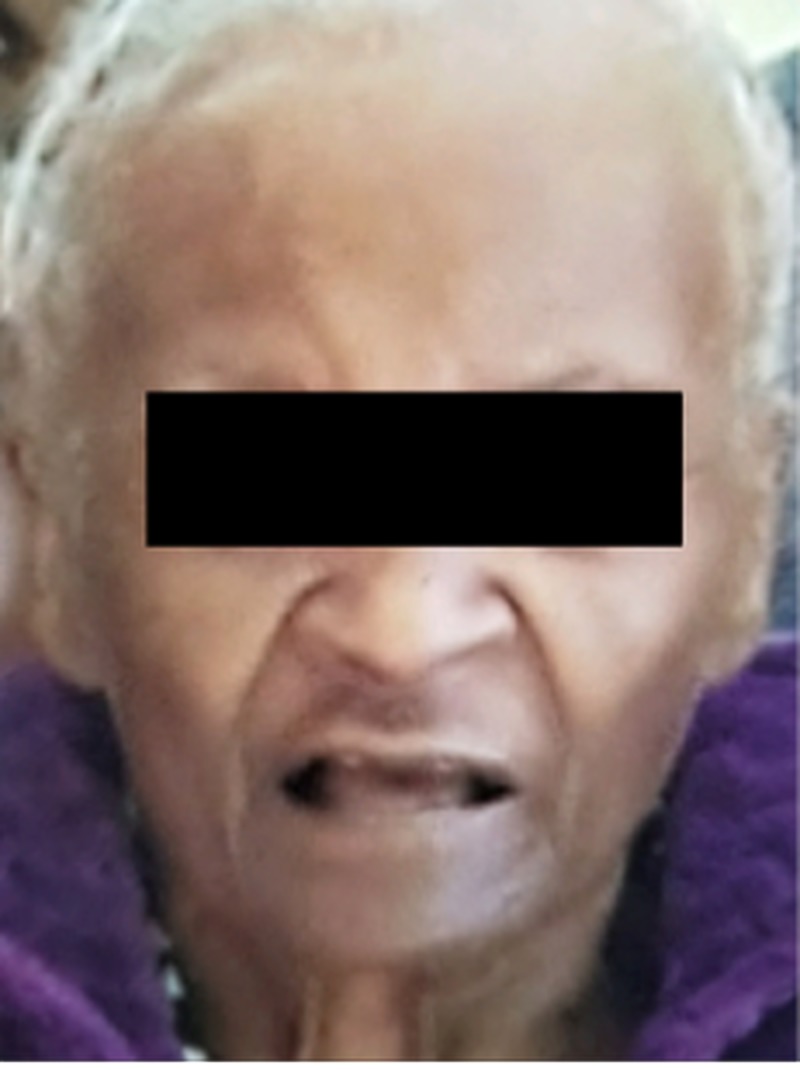
Picture taken a month before starting treatment with capecitabine.

Most striking was the marked discoloration of the patient’s face, especially on the forehead and around the nose region (Figure 7). For comparison, the picture taken before the start of chemotherapy is also shown below (Figure 8).

**Figure 9:**
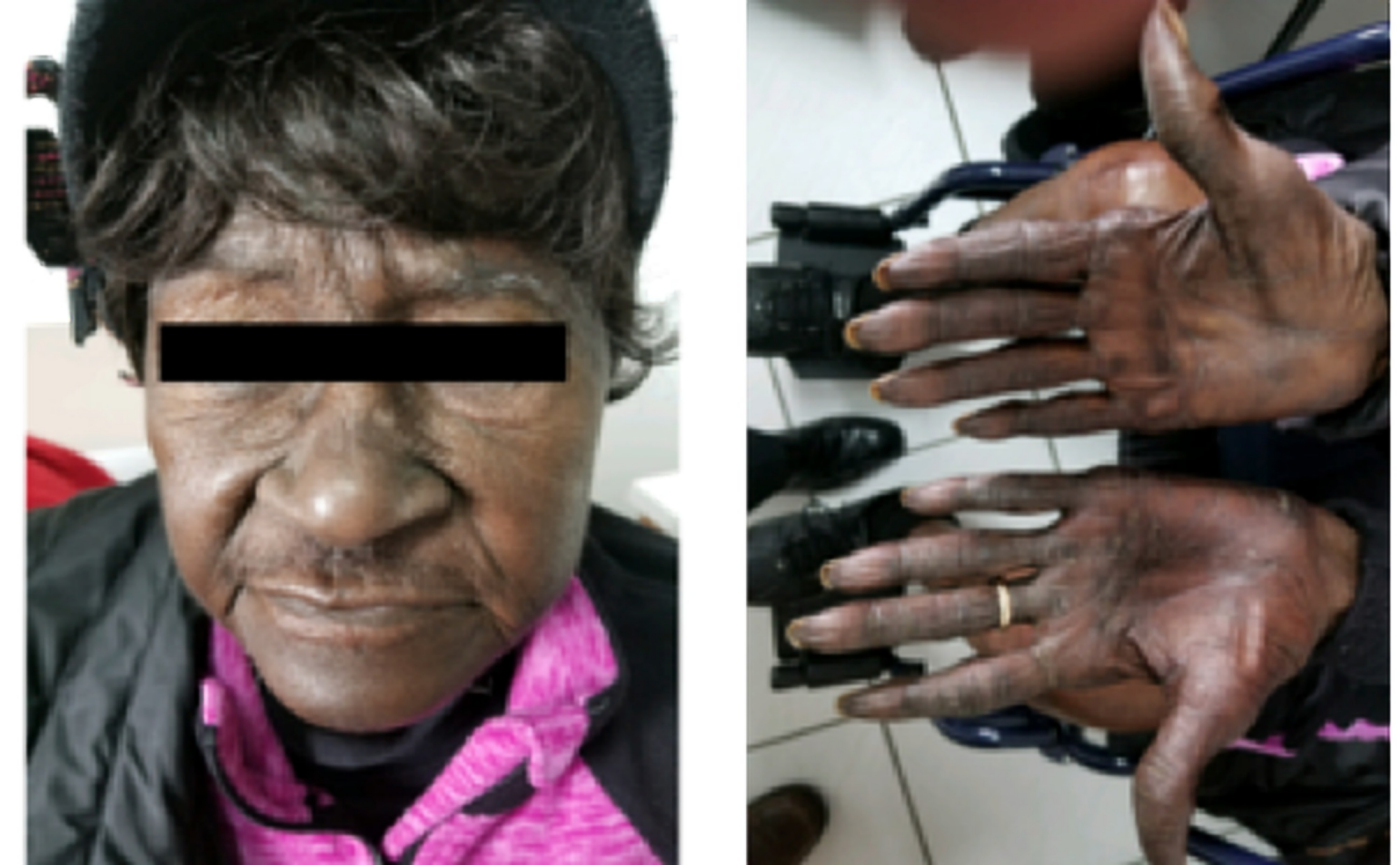
Picture taken one week after discontinuing the capecitabine.

The medication was stopped. One week after discontinuing the medication, the discoloration improved on the patient’s face and hands (Figure 9).

## Discussion

Hand-Foot syndrome is also known as palmar-plantar erythrodysesthesia (PPE). It can be categorized into three grades, as set by the National Cancer Institute Cancer Therapy Evaluation Program [2]. Grade 1 is the least severe and presents with erythema, swelling, numbness, and paresthesias, most commonly of the hands but can also involve the feet. The syndrome can progress to Grade 2, which is associated with pain that affects the activities of daily living, and finally to Grade 3, which involves blisters, moist desquamation, ulceration, and severe pain [2-4]. It has been reported that patients suffering from HFS have also presented with hyperpigmentation of the hands, feet, oral mucosa, and, in our case, the skin of the face, though this is not a part of the grading criteria [3].

Capecitabine is known to cause HFS in approximately 50% of patients [4]. The mechanism of this is still unclear, though several theories have been put forth to explain the symptoms of HFS. One possible explanation is that capecitabine causes HFS through mitochondrial dysfunction by activating caspase-dependent apoptosis, which stimulates the death of keratinocytes [5]. Another proposed mechanism is the local trauma to the small vessels from the mechanical stress of daily activities, which allows the drug to leak into the surrounding tissue, causing damage [1]. Yet another theory states that capecitabine may be eliminated by the eccrine glands, present in large numbers in the hands and feet, leading to damage to the local tissue [2]. Even though it is still unclear what is causing capecitabine-induced HFS, the most common way to manage the syndrome is to decrease or completely discontinue the drug and apply topical emollients and creams until the symptoms resolve [6].

Hyperpigmentation has been known to occur at the same time as HFS, though it is unclear whether the two things are related or are separate entities that occur simultaneously. Some reports argue that because hyperpigmentation is so often associated with early HFS, it should be considered part of the initial presentation of Grade 1 Hand-Foot syndrome [4]. Others have reported that capecitabine-associated hyperpigmentation may be a separate symptom from HFS with a different inciting mechanism, though the mechanism is unclear. One case reported the hyperpigmentation of the sole of the foot, presenting without signs of HFS, further supporting the argument that these are two separate entities [7]. Another report discusses a patient with capecitabine-associated Hand-Foot syndrome, who presented with hyperpigmentation of hands and feet and the oral mucosa, suggesting that the presence of oral hyperpigmentation may also point to two separate symptoms [3]. Our patient’s unusual presentation of hyperpigmentation of the face as well as the hand, feet, and oral mucosa may further support that hyperpigmentation has its own mechanism, separate from HFS.

## Conclusions

Hyperpigmentation does not cause physical pain to the patient but when it affects the face and other cosmetic areas, it may cause undue stress to the patient, which could negatively affect their overall treatment. In addition, if hyperpigmentation consistently occurs before the onset of Hand-Foot syndrome, patients can be advised to look out for this symptom so that action can be taken to prevent the potentially debilitating effects of HFS. Thus, research should be done in the future to better understand the relationship of hyperpigmentation to Hand-Foot syndrome and the underlying mechanism causing the hyperpigmentation in the hope that this can be prevented.

## References

[1] Almeida da Cruz L, Hoff PM, Ferrari CL, Riechelmann RS (2012). Unilateral hand-foot syndrome: does it take sides? Case report and literature review. Clin Colorectal Cancer.

[2] Sanghi S, Grewal RS, Vasudevan B, Nagure A (2013). Capecitabine induced hand-foot syndrome: report of two cases. Med J Armed Forces India.

[3] Vasudevan B (2010). An unusual case of capecitabine hyperpigmentation: is hyperpigmentation a part of hand-foot syndrome or a separate entity?. Indian J Pharmacol.

[4] Narasimhan P, Narasimhan S, Hitti IF, Rachita M (2004). Serious hand-and-foot syndrome in black patients treated with capecitabine: report of 3 cases and review of the literature. Cutis.

[5] Chen M, Chen J, Peng X (2017). The contribution of keratinocytes in capecitabine-stimulated hand-foot syndrome. Environ Toxicol Pharmacol.

[6] Lassere Y, Hoff P (2004). Management of hand-foot syndrome in patients treated with capecitabine (Xeloda). Eur J Oncol Nurs.

[7] Tognetti L, Fimiani M, Rubegni P (2018). Benign dermoscopic parallel ridge pattern in plantar hyperpigmentation due to capecitabine. Dermatol Pract Concept.

